# Bilateral optic neuritis as the presenting sign of post SARS-CoV-2 acute disseminated encephalomyelitis

**DOI:** 10.1016/j.ajoc.2022.101273

**Published:** 2022-02-04

**Authors:** Tommaso Rossi, Giovanni Novi, Isabella D'Agostino, Luca di Cello, Maria Romana Soldati, Serena Telani, Guido Ripandelli

**Affiliations:** a IRCCS Ospedale Policlinico San Martino, Department of Ophthalmology, Genoa, Italy; b IRCCS Ospedale Policlinico San Martino Department of Neurosciences, Genoa, Italy; c IRCCS Fondazione G.B. Bietti ONLUS, Rome, Italy

**Keywords:** COVID-19, Bilateral optic neuritis, Acute disseminated encephalomyelitis

## Abstract

**Purpose:**

to report a case of Acute Disseminated EncephaloMyelitis (ADEM) occurring after documented SARS-Cov2 infection and flu-like disease.

**Observation:**

A 59-years-old woman presented with progressive visual loss and right leg paresthesia started 6 days earlier when CT scan excluded abnormalities. Visual acuity was OU hand motion with bilateral slow pupillary response and unremarkable ocular extrinsic motility while visual field testing showed diffuse bilateral sensitivity reduction. The patient had also right leg paresthesia and reported a 2-weeks flu-like syndrome 15 days earlier, with nausea, diarrhea, anosmia, ageusia, cough. Brain Magnetic Resonance Imaging revealed bilateral optic nerve enhancement, multiple brain and spine lesions. SARS-CoV-2 PCR tested negative on nasal swab and positive on cerebrospinal fluid. Patient's serum tested positive for anti-SARS-CoV-2 IgG, negative for anti-aquaporin-4 and anti-myelin oligodendrocyte glycoprotein antibodies. A diagnosis of suspect ADEM *post* SARS-CoV-2 infection was made and treatment with high dose intravenous methylprednisolone (with subsequent prednisone tapering) and immunoglobulins started. Ten days later vision improved to 20/30 RE and 20/25 LE and 3 months later to 20/20.

**Conclusion and Importance:**

ADEM may ensue after SARS-CoV-2 virus infection. High suspicious index and prompt aggressive treatment may result in complete vision restauration.

## 1 Case report

A 59-year-old woman presented with 6-day history of bilateral painful, severe vision loss and right leg paresthesia one month following flu-like syndrome, anosmia, ageusia, cough, and diarrhea. Medical history included vitiligo, Hashimoto's thyroiditis and Monoclonal Gammopathy of Undetermined Significance diagnosed 3 years earlier managed through clinical follow-up.

On presentation visual acuity was hand motion in both eyes, pupillary light responses were sluggish in both eyes with no relative afferent pupillary defect. Eye movement was full but associated with pain in all directions of gaze. Static perimetry revealed severe visual field defect in both eyes and colour testing proved impossible. Anterior and posterior segment examination, including optic nerves, was unremarkable as well as and ocular coherence tomography. Neurological examination showed ageusia with anosmia, irritability, headache, and a sensory-motor thoracic spinal syndrome with lower-limbs left-sided hyperreflexia with Babinski sign and right-sided sensory impairment.

Neurological symptoms and signs were highly suggestive of encephalopathy with optic nerves and spinal cord involvement.

The patient reported a flu-like syndrome lasting two weeks with nausea, diarrhea, anosmia, ageusia, cough (without dyspnoea), that started in about a month earlier and resolved after 15 days, except for anosmia and ageusia that persisted. She also referred right limb paresthesia starting 4 days earlier that she imputed to her long standing slipped L5-S1 disc.

**Fig. 1:**
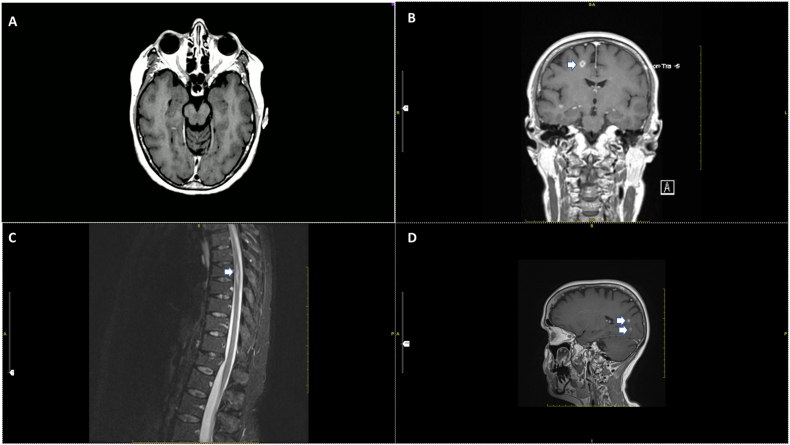
MRI of the brain (a–b) showing multiple post-Gadolinium T_1_-weighted (T1w) hyperintense lesions, some with “open ring” pattern on a a) Post-Gadolinium-T1w sequence of the brain in the axial plane showing bilateral optic nerves enhancement; b) coronal plane;. MRI of the spinal cord on a T_2_-weighted sequence showing an hyperintense spindle D7-D8 lesion.d) sagittal plane.

Upon admission patient underwent complete blood test (unremarkable, except for ESR that was 51 mm/h (normal values 0–20 mm/h) and positive anti-nucleocapsid severe acute respiratory syndrome-Coronavirus-2 (SARS-CoV-2) IgG) and nasal swab for SARS-CoV-2 polymerase chain reaction (PCR), that tested negative. Brain and spinal MRI were then performed (Fig. 1) and revealed bilateral optic nerve enhancement, multiple T_1_-weighted post-gadolinium enhancing brain and spine MRI showed a D7-D8 spinal cord lesion. A spinal tap was then performed and showed lymphocytic pleocytosis with 22 cells/mm^3^ (0–5 cells/mm3), with 95% CD3^+^ T cells, and increased CD4^+^ to CD8^+^ of 7:1 ratio (reference range: 1.2–2:1), with mild hyperproteinorrachia (452 mg/L; normal: 150–450 mg/L). SARS-CoV-2 PCR test of the cerebrospinal fluid (CSF) was positive, probably reflecting a delayed clearance of the virus in the central nervous system (CNS). Patient's serum and CSF tested negative for anti-aquaporin-4 (AQP-4) and anti-myelin oligodendrocyte glycoprotein (MOG) antibodies (ab).

Based on history, MRI finding, and evidence of SARS-CoV-2 infection, patient was diagnosed with Acute Disseminated EncephaloMyelitis (ADEM) associated with SARS-CoV-2 infection and patient treated with intravenous methylprednisolone 1 g/day for five days followed by concomitant intravenous immunoglobulins (2 g/kg in 5 days) and oral prednisone 75 mg/day (that was subsequently tapered).

Ten days later vision improved to 20/30 RE and 20/25 LE with colour vision deficiency (1/13 Ishihara plates) and impaired visual field with diffuse reduction of differential sensitivity (OD Mean Deviation (MD) −18 dB; OS 16.45 dB). Visual evoked potential showed prolonged P100 latency in both eyes (right eye 114 ms, left eye 120 ms). The number of gadolinium-enhancing lesions reduced in MRI and the patient was discharged with oral prednisone tapering.

On May 12th the patients returned to 20/20 vision OU, visual field further improved with OD MD -4,07 dB and OS MD -3.39 and correctly identified 12/12 Ishiara Plates. On July 2020 vision remained stable at 20/20 OU, MD improved to Od −1.81 dB and OS -1.79 dB. Unfortunately, the patient developed signs of femoral head necrosis secondary to steroid therapy that was discontinued and started on cyclosporine treatment.

## 2 Discussion and conclusion

The patient we report presented with subacute, ingravescent bilateral visual loss and lower limb paresthesia: differential diagnosis included bilateral optic neuritis, in the setting of Multiple Sclerosis (MS) or Neuromyelitis Optica Spectrum Disorder (NMOSD), giant cell arteritis, stroke or CNS tumour. All the above conditions can induce rapidly worsening vision loss although the simultaneous bilateral presentation is rare. However, absence of oligoclonal bands, of anti-AQ4 and anti-MOG ab make MS and NMOSD diagnosis unlikely.^1^ However, presence of lymphocytic pleocytosis with increased CD4 to CD8 radio supports the hypothesis of an autoimmune CNS disease. Furthermore, absence of high ESR and PCR values and of funduscopic signs of optic neuropathy exclude a giant cell arteritis diagnosis. Stroke and CNS tumours were also excluded by CT and MRI scans. Bilateral retrobulbar optic neuritis is possible but infrequent and allows ambulation while arteritis is usually asynchronous and associated to very high ESR and PCR values.

**Fig. 2:**
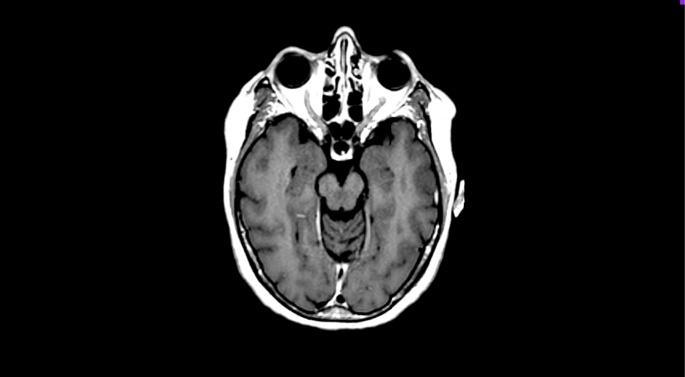
Post-Gadolinium (Gd) T1 weighted (T1w) sequence of the brain in the axial plane showing bilateral optic nerves enhancement.

ADEM is an immune-mediated demyelinating CNS disorder occurring 2–4 weeks after viral infection^2^, characterized by acute polyfocal neurologic deficits progressing within 5–7 days. Our patient's MRI showed multiple lesions throughout the brain, spine (Fig. 1), and optic nerves (Fig. 2), justifying visual field loss with central scotoma, and neurological abnormalities. MRI lesions were highly suggestive of ADEM demyelination due to the characteristic synchronous pattern involving the CNS white matter with hyperintensities in post-Gadolinium-T_1_-weighted and T_2_-weighted sequences. It should be pointed out that ADEM is always a presumptive diagnosis based on the exclusion of other likely possibilities that, in our case, could have been triggered by SARS-CoV-2 infection. In fact, our patient reported the presence of flu-like symptoms associated with anosmia and ageusia that preceded the onset of neurological symptoms. This hypothesis is also supported by the presence of anti-SARS-CoV-2-nucleocapsid IgG ab.

Vitiligo and Hashimoto's disease co-morbidity suggest auto-immune diathesis and multiple demyelinating lesions may indeed represent ADEM although this is a diagnosis of exclusion. SARS-CoV-2 involvement of the CNS is reported in 25% of COVID-19 patients^3^ showing headache, dizziness, strokes, Guillain-Barrè Syndrome and meningitis. Coronaviruses have been associated to ADEM^4^ and cerebrospinal fluid PCR positivity to SARS-CoV-2 have been described^5^ although no ADEM specifically associated to SARS-CoV-2 cases have been reported to the best of our knowledge.

## Patient consent

Written consent to publish this case has not been obtained. This report does not contain any personal identifying information.

## Funding

No funding or grant support.

## Authorship

All authors attest that they meet the current ICMJE criteria for Authorship.

## Financial disclosure

None of the authors have any financial interest in the subject.

## Declaration of competing interest

None of the Authors has anything to disclose.
